# Three-dimensional versus two-dimensional high-definition laparoscopy in transabdominal preperitoneal inguinal hernia repair: a prospective randomized controlled study

**DOI:** 10.1007/s00464-019-07266-z

**Published:** 2019-11-21

**Authors:** Hanna E. Koppatz, Jukka I. Harju, Jukka E. Sirén, Panu J. Mentula, Tom M. Scheinin, Ville J. Sallinen

**Affiliations:** 1 Department of Abdominal Surgery, Helsinki University Hospital and University of Helsinki, Haartmaninkatu 4, 00029 Helsinki, Finland; 2 Department of Transplantation and Liver Surgery, Helsinki University Hospital and University of Helsinki, Helsinki, Finland

**Keywords:** Laparoscopic, TAPP, 3D, 2D

## Abstract

**Background:**

Three-dimensional (3D) laparoscopy improves technical efficacy in laboratory environment, but evidence for clinical benefit is lacking. The aim of this study was to determine whether the 3D laparoscopy is beneficial in transabdominal preperitoneal laparoscopic inguinal hernia repair (TAPP).

**Method:**

In this prospective, single-blinded, single-center, superior randomized trial, patients scheduled for TAPP were randomly allocated to either 3D or two-dimensional (2D) TAPP laparoscopic approaches. Patients were excluded if secondary operation was planned, the risk of conversion was high, or the surgeon had less than five previous 3D laparoscopic procedures. Patients were operated on by 13 residents and 3 attendings. The primary endpoint was operation time. The study was registered in ClinicalTrials.gov (NCT02367573).

**Results:**

Total 278 patients were randomized between 5th February 2015 and 23rd October 2017. Median operation time was shorter in the 3D group (56.0 min vs. 68.0 min, *p* < 0.001). 10 (8%) patients in 3D group and 6 (5%) patients in 2D group had clinically significant complications (Clavien–Dindo 2 or higher) (*p* = 0.440). Rate of hernia recurrence was similar between groups at 1-year follow-up. In the subgroup analyses, operation time was shorter in 3D laparoscopy among attendings, residents, female surgeons, surgeons with perfect stereovision, surgeons with > 50 3D laparoscopic procedures, surgeons with any experience in TAPP, patients with body mass indices < 30, and bilateral inguinal hernia repairs.

**Conclusion:**

3D laparoscopy is beneficial and shortens operation time but does not affect safety or long-term outcomes of TAPP.

Three-dimensional (3D) laparoscopy is one of the latest innovations gaining popularity in laparoscopy surgery and has been claimed to improve surgical efficacy, reduce errors, increase spatial awareness, and reduce time to complete surgical tasks in laboratory environment [1–9]. Recently, several randomized controlled studies performed in clinical environment have tried to find out whether these advantages of 3D laparoscopy found in laboratory environment are transferable to clinical settings. These trials have reported conflicting results; some report benefits of 3D laparoscopy [10–14], while others did not find advantages in using 3D over conventional 2D laparoscopy [11, 12, 15–17]. Unfortunately, most of the earlier trials have been limited by small sample size and small number of surgeons involved.

Transabdominal preperitoneal inguinal hernia repair (TAPP) is one of the most common laparoscopic surgical procedure carried out as a day case surgery. Approximately, 115,000 such operations are carried out annually in the USA [18], and the rate is likely to increase in the future. There are, to our knowledge, no randomized controlled trials comparing 3D laparoscopy to 2D laparoscopy in TAPP. TAPP is a more complex procedure than other procedures carried out as a day case surgery, such as laparoscopic cholecystectomy, where no benefit of 3D was found [15]. In order to justify the more expensive 3D laparoscopes, there must be evidence for benefit and safety. Based on IDEAL framework which states that no surgical innovation should occur without evaluation [19], we designed a randomized controlled trial to assess surgical efficacy and safety of 3D laparoscopy in transabdominal preperitoneal inguinal hernia repair.

## Materials and methods

This was a randomized controlled trial conducted in the day surgery department of an academic teaching hospital (Helsinki University Hospital) which functions as a secondary referral center for 1.2 million inhabitants and tertiary referral center for 1.9 million inhabitants. Patients scheduled for TAPP in an operating room equipped with 3D laparoscopic instrumentation were assessed for eligibility. Patients were excluded if a secondary operation in addition to TAPP was planned, if conversion to laparotomy was deemed likely (such as history of numerous abdominal operations, or peritonitis), or if the surgeon had an inadequate experience in 3D laparoscopy (defined as less than five 3D laparoscopic procedures). The limit was based on an earlier report indicating that the learning curve for 3D laparoscopy included five procedures [20]. The overall study design was similar to earlier randomized controlled trial comparing 3D to 2D laparoscopy in cholecystectomy by our research group [15].

### Power calculation and randomization

Operation time was chosen as the primary outcome measure. Secondary outcomes were conversion rate, intraoperative complications, postoperative complications (Clavien–Dindo), need for hospital stay, estimated blood loss, hospital readmission, mortality, and operation room time. In order to calculate power, the operation times of the TAPP procedures performed in the department were extracted from the electronic operating room log. The patients for inclusion in this calculation were searched using NCSP (Nordic Classification of Surgical Procedures)-code JAB11 (TAPP) as a primary procedure in the year 2013. Cases with a secondary procedure code were excluded. The mean operative time for all TAPP procedures was 62.0 min (SD 29.4 min, *n* = 126): it was 50.4 min (SD 18.5 min, *n* = 78) for unilateral TAPP and 80.8 min (SD 33.8 min, *n* = 48) for bilateral TAPP. The study aimed to show 10 min difference in mean operative time (62 vs 52 min). Standard deviation was assumed to be 29.4 in both 2D and 3D groups. Based on two-tailed power calculation with 80% power, 0.05 alpha, 1:1 allocation, 274 patients were needed to be included in the trial to show this difference. A block randomization with a 1:1 allocation and a randomly varied block size of 4 to 6 was generated using Blockrand 1.1 package with R Statistical Software. Paper indicating the allocated group were enclosed in sequentially numbered, opaque, sealed envelopes. At the time of inclusion, the envelopes were opened sequentially by the operating surgeon prior the operation. The patient was blinded to their randomization group.

### Instrumentation and interventions

Wolf^®^ (Richard Wolf Medical Instruments^®^, Chicago, Illinois, USA) laparoscopic HD device with a non-deflectable 30° scopes was used for all the operations. The device can be set into 3D or 2D mode. For the 3D group cases, the device was set to 3D, and for the 2D group it was set to 2D. The surgeons were allowed to switch from 3D to 2D if needed (e.g., during trocar insertion), but switching from 2D to 3D in the 2D group was not allowed. Adherence to the randomized group (2D or 3D) was assessed by a case report form which the surgeon filled after the operation. During the 3D laparoscopy, the surgeons wore passive polarizing glasses, whereas no extra glasses were worn in the 2D cases. The surgeons were allowed to define the proper viewing position for themselves to avoid any disturbances in vision.

Residents performing the operations had at least 3 years of surgical experience. An attending was always present when a resident was the main surgeon. The number of previous procedures (TAPP, or 3D procedures in general) were recorded for each surgeon (classified as < 20, > 20, or > 50 previous cases for TAPP; < 10, > 10, or > 50 previous cases for 3D laparoscopic procedures in general). The subjective satisfaction of each surgeon was collected based on a 0–10 Likert scale score, and the surgeons were free to express comments or concerns regarding the laparoscope in free text form after the operation. The stereo acuity was measured using the Randot^®^ Stereotest (Stereo Optical, Chicago, Illinois, USA), but surgeons were neither selected nor excluded based on the test. The Randot test consists of ten sets of three circles, one of which has a crossed disparity and appears to be closer. Between the sets, the disparity decreases from 400 to 20 s of arc. If the surgeon could not distinguish the differences between the sets, he/she was considered stereo blind (0 points). Otherwise, the level of stereopsis was defined as the last circle identified correctly. The level of perfect stereopsis was defined to 20 s of arc (10 points).

The operation time was defined as the time from the first incision until closure of the skin. First 12 mm trocars were inserted umbilically and in the left or right lower abdomen, depending on side of the hernia. One 5 mm trocar was inserted in side opposite of the hernia. In cases of bilateral hernias, a 12 mm trocar was used in place of the 5 mm trocar. The peritoneum was opened cranial to the hernia site. Peritoneum was taken down and the hernia was reduced by blunt and sharp dissection. A polypropylene mesh (Biomesh^®^ P1, Cousin Biotech, Wervicq-Sud, France) was placed to cover the hernia defect and attached using glue (LiquiBand^®^Fix8™, Advanced Medical Solutions, Devon, UK). The mesh was then covered by a peritoneal flap by suturing the peritoneum back to the original position using absorbable suture (V-Lock™180, Covidien, Mansfield, USA). If the peritoneum was tattered, the Dynamesh^®^ IPOM was used. Fascia in 12 mm port sites was closed. Surgeons were allowed to deviate from these routines if deemed necessary for patient safety.

Thirty-day complications were assessed from the electronic medical records, and the patients were also contacted by phone 30 days after the operation. In cases where the patient did not respond to phone calls, they were contacted by letter. Patients were contacted again after 1 year by a letter for long-term follow-up regarding recurrent hernia and pain in the groin area. If they did not respond, the letter was re-posted and thereafter patients were contacted by phone. Recurrences were recorded only if they had been diagnosed by a doctor. If patient reported a possible hernia not diagnosed by a doctor, the patients were contacted for further information about possible hernia. If based on this contact a hernia seemed possible, the patient was physically examined for recurrent hernia. The level of pain at 1-year follow-up was reported in our results if the patient had the experience daily or at least weekly.

### Statistical analysis

Continuous variables were compared between groups using the Mann–Whitney *U* test and *p*-values < 0.05 were considered statistically significant. Subgroups analyses based on the sex of the surgeon, the surgeon’s level of experience, resident versus attending status, and stereovision were specified a priori. The statistical analyses were performed using SPSS^®^ version 22 (IBM, Armonk, NY). Primary outcome was analyzed using modified intention to treat principle in which all randomized patients who underwent TAPP were included in the analysis. Adjusted multivariate analyses were performed using linear regression with log-transformed primary outcome.

The study was approved by the institutional review board and the ethical board of Helsinki University Hospital. All patients gave informed written consent to participate in the study. The trial was registered in ClinicalTrials.gov before commencement (NCT02367573).

## Results

**Fig. 1:**
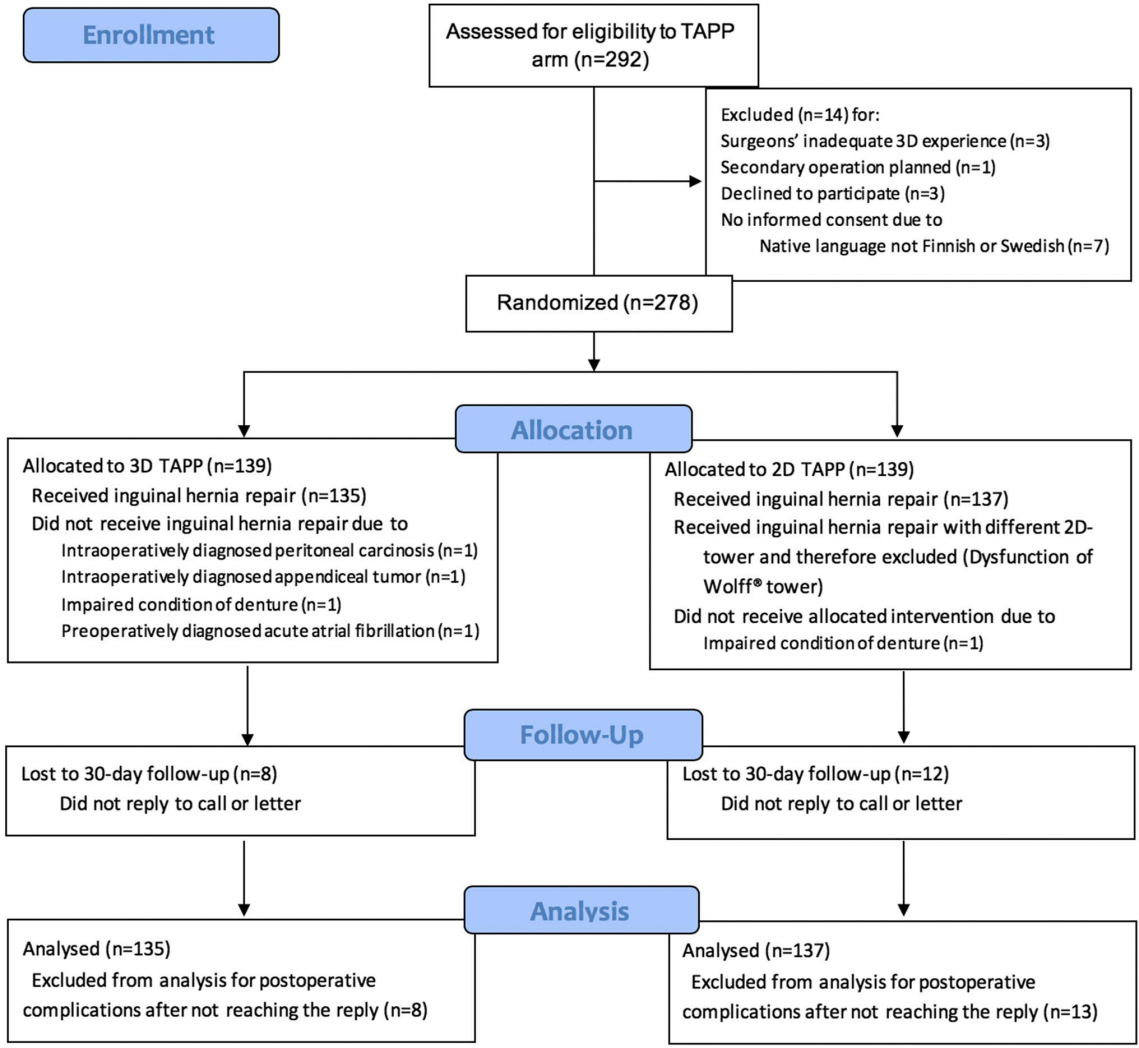
Flow chart of patient selection, randomization, and follow-up

A total of 292 patients were assessed for eligibility between 5th February 2015 and 23rd October 2017, 14 patients were excluded, and 278 patients were randomly allocated to either 3D or 2D laparoscopic TAPP (Fig. 1). All randomized patients were included in the primary outcome analysis, except those that did not undergo TAPP surgery (four patients in 3D group, one patient in 2D group, Fig. 1).

The patients’ basic characteristics, types of hernia (uni- or bilateral, primary or recurrent), and the characteristics of the surgeons were similar between the 3D and 2D groups (Table 1). Operations were performed by 13 residents (7 women) and 3 attendings (1 woman) with variable experience in TAPP and 3D laparoscopy (Table 1). Two surgeons reported double vision and nausea when operating with 3D and five surgeons reported more difficulties with peritoneal suture in the 2D group. One operation was converted from 3D to 2D due to a technical dysfunction with the 3D equipment.

**Table 1:** Basic characteristic of patients undergoing transabdominal preperitoneal inguinal hernia repair and of the surgeons operating on them

	3D (*n* = 135)	2D (*n* = 137)
Age, median; IqR	57.9; 39.6–65.5	55.8; 45.2–63.3
BMI, median; IqR	24.4; 22.5–28.3	24.7; 22.8–28.7
Male patient	98 (72.6%)	106 (77.9%)
ASA classification, *n* (%)		
1	61 (45.2%)	62 (45.6%)
2	65 (48.1%)	64 (46.7%)
3	8 (6.7%)	11 (8.0%)
Charlson comorbidity index, *n* (%)		
0	118 (87.4%)	127 (92.7%)
1	17 (12.6%)	10 (7.3%)
History of earlier abdominal operations, *n* (%)		
Open	13 (9.6%)	13 (9.5%)
Laparoscopic	10 (7.4%)	7 (5.1%)
Indication, *n* (%)		
Unilateral primary hernia	50 (37.0%)	44 (32.4%)
Bilateral primary hernia	49 (36.3%)	63 (46.3%)
Unilateral recurrent hernia	27 (20.0%)	19 (14.0%)
Bilateral recurrent hernia	3 (2.2%)	5 (2.9%)
Bilateral, primary + recurrent hernia	3 (2.2%)	4 (2.9%)
Other (inguinal pain or “Sportman’s hernia”)	3 (2.2%)	2 (1.5%)
Surgeon, *n* (%)		
Man	126 (93.3%)	116 (84.7%)
Attending	108 (80.0%)	97 (70.8%)
Resident	27 (20.0%)	40 (29.2%)
Surgeon experience in TAPP, cases (%)		
< 20	25 (18.5%)	39 (28.5%)
20–50	5 (3.7%)	5 (3.6%)
> 50	105 (77.8%)	93 (67.9%)
Surgeon experience in 3D laparoscopy, cases (%)		
5–10	9 (6.7%)	12 (8.8%)
10–50	28 (20.7%)	35 (25.5%)
> 50	98 (72.6%)	90 (65.7%)
Surgeon stereo acuity, stereopsis 10, *n* (%)	74 (70.5%)	80 (76.9%)

*2D* two-dimensional, *3D* three-dimensional, *BMI* body mass index, *ASA* The American Society of Anesthesiologists physical status classification, *TAPP* transabdominal preperitoneal inguinal hernia repair

The primary outcome, operation time, was on average 12 min shorter in the 3D group compared to the 2D group (median 56 min (interquartile range (IQR) 43–71) versus 68 min (IQR 50–86), *p* < 0.001) (Table 2). In the subgroup analyses, operation time was shorter in the 3D group (compared to 2D group) among attendings, residents, female residents (but not in male residents), surgeons with perfect stereovision (but not in surgeons with imperfect stereovision), surgeons with > 50 3D laparoscopic procedures, surgeons with any level of experience in TAPP, patients with body mass index less than 30, and bilateral (but not in unilateral) hernia repairs (Table 3). In order to adjust effect of confounding factors including hernia laterality, surgeon’s sex, attending/resident, surgeon expertise in TAPP and 3D, and stereopsis, a linear regression analysis was performed. After adjustment, 3D laparoscopy had a statistically significant effect on operation time (regression coefficient − 0.037, 95% CI − 0.062 to − 0.012) *p* = 0.004.

**Table 2:** Outcome measures after transabdominal preperitoneal inguinal hernia repair

	3D (*n* = 135)	2D (*n* = 137)	*p*-value
Operating room time, min, median; IqR	125.0; 107.0–144.5	139.0; 115.3–157.8	0.001
Operation time, min, median; IqR	56.0; 43.0–71.0	68.0; 50.3–85.8	< 0.001
Estimated blood loss, ml, mean (SD)	1.3 (3.4)	1.8 (5.5)	0.345
Intraoperative complications, *n* (%)			
None	123 (91.1%)	130 (94.9%)	0.356
Bleeding (intra-abdominal)	3 (2.2%)	2 (1.5%)	
Small bowel serosa injury	1 (0.7%)	0	
Peritoneal tear	8 (5.9%)	5 (3.6%)	
Postoperative complication, *n* (%)^a^			
Total	32 (25.2%)	24 (19.2%)	0.290
CD I	23 (18.1%)	20 (16.0%)	0.738
Abnormal pain	10 (7.4%)	11 (8.0%)	
Scrotal hematoma	8 (5.9%)	3 (2.3%)	
Bleeding (abdominal wall)	1 (0.7%)	1 (0.7%)	
Urinary retention	1 (0.7%)	0	
Gastroenteritis	1 (0.7%)	1 (0.7%)	
Other	5 (3.7%)	4 (2.9%)	
CD II	10 (7.9%)	6 (4.8%)	0.440
Bowel occlusion	0	2 (1.4%)	
Wound infection	6 (4.4%)	1 (0.7%)^b^	
Urinary tract infection	1 (0.7%)	1 (0.7%)	
Epididymo-orchitis	1 (0.7%)	0	
Prostatitis	1 (0.7%)	0	
Pneumonia	1 (0.7%)	2 (1.4%)	
CD IIIb			0.496
Small bowel perforation	0	1 (0.7%)^b^	
Satisfaction with laparoscopic view, attendings, median; IqR	10; 10	10; 9–10	0.001
Satisfaction with laparoscopic view, residents, median; IqR	9; 7.5–9	8; 7–9	0.150

*CD* Clavien–Dindo classification for postoperative complications within 30 days, *IqR* interquartile range, *SD* standard deviation, *TAPP* transabdominal preperitoneal inguinal hernia repair

^a^Eight patients had more than one complication, ^b^two complications in one patient

**Table 3:** Subgroup analysis of operation time for transabdominal preperitoneal inguinal hernia repair

Subgroup	3D min; IqR (*N*)	2D min; IqR (*N*)	*p*-value
Surgeon status			
Attendings	50.5; 42.0–65.0 (108)	63.0; 44.5–80.0 (97)	0.031
Residents	67.0; 61.0–87.0 (27)	84.0; 69.0–102.3 (39)	0.008
Sex^a^			
Male resident	70.0; 64.0–89.0 (19)	87.0; 62.0–114.0 (21)	0.307
Female resident	65.5; 53.8–83.3 (8)	84.0; 72.0–97.0 (19)	0.047
Stereovision^a^			
Stereopsis 10	62.5; 52.3–70.3 (8)	72.0; 63.0–88.5 (13)	0.037
Stereopsis ≤ 9	81.0; 64.0–90.0 (19)	89.0; 74.0–114.0 (27)	0.144
3D experience			
≤ 50	67.0; 50.0–85.0 (37)	75.0; 61.0–93.0 (47)	0.075
> 50	53.0; 43.0–65.0 (98)	64.5; 47.5–81.3 (90)	0.002
TAPP experience			
≤ 50	71.0; 63.3–87.5 (30)	84.5; 72.0–102.3 (44)	0.035
> 50	50.0; 42.0–65.0 (105)	61.0; 44.0–77.0 (93)	0.01
Patient BMI			
≤ 25	56.0; 43.0–72.0 (79)	68.0; 51.0–86.0 (75)	0.005
25–30	57.0; 44.0–68.0 (51)	67.0; 49.0–86.5 (57)	0.032
> 30	43.0; 39.0–107.5 (5)	70.0; 64.0–111.5 (5)	0.421
Hernia type			
Unilateral	47.0; 40.0–56.0 (78)	51.0; 40.0–64.0 (67)	0.125
Bilateral	68.0; 62.0–84.0 (57)	83.0; 70.0–94.0 (70)	0.001

*2D* two-dimensional, *3D* three-dimensional, *BMI* body mass index, *IqR* interquartile range, *TAPP* transabdominal preperitoneal inguinal hernia repair

^a^Only residents

There were no conversions to open surgery in either group. Intraoperative complications were infrequent, minor, and without differences between the 3D and 2D groups (Table 2). Most frequent intraoperative complication was a peritoneal torn due to difficult hernia preparation or adhesions. One serosal injury to the small bowel occurred in the 3D group and was repaired by suturing without further postoperative complication. Three patients (1.2%) were readmitted within 30 days in 2D group due to bowel occlusion (*N* = 2) and bowel perforation (*N* = 1). No readmissions occurred in 3D group. Twenty-six (10.3%) patients in 3D group and 21 (8.3%) patients in 2D group had an outpatient visit within 30 days. There was no difference in postoperative complications between the 3D and 2D groups. Almost all complications were Clavien–Dindo class 1–2, and only one Clavien–Dindo class 3 complication occurred in the 2D group (Table 2): The fascia at the trocar insertion site was closed with a suture that was inadvertently passed through small bowel wall. This caused small bowel perforation which was repaired by suturing via laparotomy. No Clavien–Dindo grade 4 or 5 complications occurred.

None of the patients died within 90 days after the operation. Although all patients were scheduled as day case surgery, 12 patients (8.8%) in the 3D group and 15 patients (11.1%) in the 2D group needed to stay overnight in the hospital, mostly due to fatigue and social issues.

224 (89.7%) out of 272 patients responded to the 1-year follow-up letter, and further 48 (17.6%) patients were contacted by phone, and only 10 (3.7%) patients could not be contacted by mail or phone. One-year follow-up rate was therefore 96.3%. No patient developed port site hernia. Three (1.1%) patients in 3D group and two (0.8%) patients in 2D group were diagnosed with recurrent inguinal hernia within 1-year follow-up (Table 4). Severe pain at 1-year follow-up was reported by two patients in 3D group and three patients in 2D group (Table 4).

**Table 4:** Pain experience and hernia recurrence at 1-month and 1-year follow-up

	3D (*n* = 135)	2D (*n* = 137)	*p*-value
Responded to 1-month questionnaire, *n* (%)	127 (94%)	125 (91%)	
Level of pain at 1-month			0.174
No pain (VAS 0-1)	76 (59.8%)	86 (68.8%)	
Mild pain (VAS 2-4)	48 (37.8%)	34 (27.2%)	
Severe pain (VAS > 5)	3 (2.4%)	5 (4.0%)	
Responded to 1-year questionnaire, *n* (%)	112 (83%)	112 (81.8%)	0.874
Level of pain at 1-year^a^			0.825
No pain or minor discomfort (VAS 0-1)	2 (1.8%)	2 (1.8%)	
Mild pain (VAS 2-4)	10 (8.9%)	8 (7.1%)	
Severe pain (VAS > 5)	2 (1.8%)	3 (2.7%)	
Hernia recurrence at 1-year	3 (1.1%)	2 (0.8%)	0.678

^a^Only patients who reported pain daily or at least weekly

## Discussion

Three-dimensional laparoscopy shortened operation times in TAPP on average 12 and operation room time on average 14 min. This increased surgical efficacy in TAPP was noted not only generally but also in several subgroups, indicating that the benefit is not restricted to only certain subgroups, but nearly all patient and surgeon groups benefit from 3D in TAPP. However, the rates of postoperative complications, intensity of pain, and the rate of hernia recurrence after 1 year were similar between 2D and 3D laparoscopy.

There is no previous research on 3D versus 2D laparoscopy regarding TAPP. TAPP is a moderately complex operation carried out in day surgery unit. It requires spatial awareness in reducing the hernia sac, dissecting the preperitoneal space, and suturing of the peritoneal flap. It is unclear which parts of the operation (dissection, mesh, placing, and suturing) are affected mostly by the 3D vision compared to 2D. In simulated settings, the temporal advantage of 3D is more demonstrable with functions demanding greater depth perception than precise tasks (such as suturing) [3, 21]. In a recent systematic review and meta-analysis, benefits of 3D in terms of operation time and complication were demonstrated particularly in operations requiring suturing [22].

Only a few studies assessing 3D laparoscopy in clinical setting have been carried out with conflicting results. 3D laparoscopy has been shown to reduce operative times in transanal pull through in children [10], radical prostatectomy [11], hiatal hernia repair [14], and mini gastric bypass [12], as well as reduce technical errors in splenic hilar lymphadenectomy [13] and warm ischemia time in partial nephrectomy [11]. On the other side, 3D laparoscopy did not provide benefits in laparoscopic cholecystectomy [15], total mesorectal excision [16], pyeloplasty [11], gastrectomy for cancer [17], or sleeve gastrectomy for obesity [12]. It could be hypothesized that a more complex operation is needed in order to gain benefits of 3D laparoscopy, and TAPP seems to be complex enough for this.

The size of the 3D laparoscope is one of its downsides. The trend in laparoscopy is towards smaller incisions, but 3D instrumentation still requires 10 mm trocars. Larger trocars might cause more port site hernias [23]. However, port site hernias did not occur during our short 30-day follow-up neither after 1 year. Only one Clavien–Dindo class IIIb complication occurred, but this was caused by inadvertently suturing the intestinal serosa with the fascia. This could have been avoided if a 5 mm laparoscope had been used, as 5 mm trocar sites need not be sutured. In our study, the inguinal hernia recurrence rate (overall 1.8%) is low compared to nationwide outcomes in the USA (a total of 140,355 laparoscopic hernia repairs, 11.9% recurrence rate) [24], but comparable to other randomized controlled trials on TAPP [25]. No difference in hernia recurrence rates could be seen between 3D and 2D groups in our trial.

In this study, the surgeons’ stereo acuity was examined using the Randot^®^ Stereotest. Two surgeons had a complete lack of stereopsis. In the subgroup analysis, surgeons with perfect stereovision had shorter operating times. There was no statistical difference in operation times between the 3D and 2D surgeries in the subgroup with imperfect stereovision, which is in line with previous studies [1, 21]. Not only the stereo acuity, but also the optimal distance to the laparoscope monitor [4] and thereafter, the precise depth perception [21] are important to achieve proper 3D laparoscopy experience. In contrast to simulated settings, we did not define the precise location of the display and the surgeons were free to define it for themselves to obtain convenient visual distance. The laparoscope is usually fixed in experimental studies, while in real clinical scenarios, such as this trial, an assistant holds and moves the camera. Of note, the operation time was longer in surgeons with imperfect stereovision compared to perfect stereovision even among 2D laparoscopy group. This might have implications in general laparoscopy training, but requires further exploration.

In subgroup analyses, the benefit of 3D was statistically significant in female surgeons, but not in male surgeons. However, the benefit in terms of reduction in operation time was almost identical between genders (17 min in males, 18.5 min in females), but the variation was larger in males making the difference statistically insignificant. It is likely that the 3D would be beneficial also in male surgeons had the cohort been larger.

This study has limitations. There was no blinding on the surgeons’ as this would have been impossible. Duration of different parts of the operation was not recorded, and thus, we can only speculate which parts would benefit most from 3D laparoscopy. However, we were able to show difference in the primary outcome, duration of surgery, which is a robust endpoint in this efficacy trial. Using only 5 mm trocars, no fascia needs to be closed and the difference of operation times might not be present. At the same time, the trial was not powered for morbidity and mortality, which are, fortunately, very rare in elective TAPP. However, our results do suggest that these are unaltered by 3D approach.

The strength of this study is the relatively large cohort size in a common day case surgery. Large numbers of surgeons with various levels of expertise were involved. The study design was very pragmatic, and the results are applicable to routine practice. The response rates at 1-month (92.6%) and 1-year (96.3%) were fairly good, and the results are considered representative of the studied cohort.

One could ask if saving 14 min of operating room time is worthwhile. One minute of operating room time is estimated to cost 36–37 USD [26]. The annual rate of laparoscopic inguinal hernia repairs are 115,000 in USA, 99,000 in Germany, 52,000 in France, 18,000 in UK, and 1100 in Finland [18, 27]. Estimated savings per year for the decreased operating room time would be 57.9 million USD in USA, 49.9 million USD in Germany, 26.2 million USD in France, 9.1 million USD in UK, and 0.6 million USD in Finland. In many institutions, 3D instrumentation has already been purchased, and in these cases, they should be used in operations, in which they provide most benefit. For example, 3D laparoscopy does not seem to provide any benefit in laparoscopic cholecystectomy [15].

## Conclusion

3D laparoscopy can increase surgical efficacy in TAPP without affecting safety or long-term outcomes.
